# Hyperadiponectinemia enhances bone formation in mice

**DOI:** 10.1186/1471-2474-12-18

**Published:** 2011-01-17

**Authors:** Yasuhiro Mitsui, Masafumi Gotoh, Nobuhiro Fukushima, Isao shirachi, Shuichi Otabe, Xiaohong Yuan, Toshihiko Hashinaga, Nobuhiko Wada, Akiko Mitsui, Tatsuhiro Yoshida, Shiro Yoshida, Kentaro Yamada, Kensei Nagata

**Affiliations:** 1 Department of Orthopedic Surgery, Kurume University, 67 Asahi-machi, Kurume, Fukuoka 830-0011, Japan; 2 Department of Orthopedic Surgery, Kurume University Medical Center, 155 Kokubu-machi, Kurume, Fukuoka 839-0863, Japan; 3 Division of Endocrinology and Metabolism, Department of Medicine, Kurume University, 67 Asahi-machi, Kurume, Fukuoka 830-0011, Japan

## Abstract

**Background:**

There is growing evidence that adiponectin, a physiologically active polypeptide secreted by adipocytes, controls not only adipose tissue but also bone metabolism. However, a role for adiponectin in bone development remains controversial.

**Methods:**

We therefore investigated the endocrine effects of adiponectin on bone metabolism using 12-week-old male transgenic (Ad-Tg) mice with significant hyperadiponectinemia overexpressing human full-length adiponectin in the liver.

**Results:**

In Ad-Tg mice, the serum level of osteocalcin was significantly increased, but the levels of RANKL, osteoprotegerin, and TRAP5b were not. Bone mass was significantly greater in Ad-Tg mice with increased bone formation. In contrast, bone resorption parameters including the number of osteoclasts and eroded surface area did not differ between Ad-Tg and their littermates.

**Conclusions:**

These findings demonstrate that hyperadiponectinemia enhances bone formation in mice.

## Background

Osteoporosis and related bone fractures are becoming increasingly common in industrial countries due to enhanced longevity [1,2]. Therefore, it is important to know the factors that regulate bone mass, and to develop effective therapeutic methods.

Adipose tissue is known to act as an energy-storing organ that secretes a variety of biologically active molecules [3]. Adiponectin is one of the adipocytokines specifically and highly expressed in adipose tissue. It has two subtypes: the full-length (predominant) form that is distributed in the circulation, and the globular form [4]. This adipocytokine is present abundantly in plasma [5] and plays important roles in the regulation of energy homeostasis and insulin sensitivity [6,7], thus exerting potent effects in various tissues.

It has been reported that the adiponectin signal is also involved in bone homeostasis, since adiponectin and its two related adiponectin receptor subtypes (AdipoR1 and AdipoR2) have been identified in osteoblasts [8-10]. Although previous studies have examined the effect of adiponectin on bone metabolisms, its physiological role remains unclear [9-13].

Recently, we have successfully established two lines of hyperadiponectinemic mice showing transgenic expression of human full-length adiponectin [14]. To test the hypothesis that the level of circulating human full-length adiponectin is positively correlated with bone metabolism, the present study focused on characterizing its endocrine effect in this strain of transgenic mice. We demonstrated that hyperadiponectinemia enhances osteogenesis through osteoblast formation.

## Methods

### Animals

Transgenic (Ad-Tg) mice overexpressing human full-length adiponectin, driven by the human serum amyloid component (SAP) promoter and with its expression limited to the liver, were generated as described previously [14]. In each analysis, male Ad-Tg and their wild type (WT) littermates generated by intercrossing between heterozygous mice were compared. All the mice were kept in plastic cages under standard laboratory conditions with a 12-h dark, 12-h light cycle, a constant temperature of 23°C, and a humidity of 48%. The mice were fed a standard rodent diet (CE-2; CLEA Japan, Inc.) containing 25.2% protein, 4.6% fat, 4.4% fiber, 6.5% ash, 3.44 Kcal/g, 2.5 IU vitamin D_3_/g, 1.09% calcium, and 0.93% phosphorus with water ad libitum. All animal experiments were performed on male mice at 12 weeks of age and were reviewed and approved by the Animal Study Committee of Kurume University.

### Skeletal morphology

There were 8 mice in each group (Lines 11 and 13 representing Ad-Tg mice, and their WT littermates): the right femur was used for bone mineral density (BMD) and bone mineral content (BMC) analysis, and the left tibia for bone histomorphometric evaluation. A total of 24 mice were sacrificed and analyzed in this study. BMD and BMC were measured in 24 femurs (8 femurs in each mouse group) using a DCS-600EX-IIIR (Aloka). Twenty-four tibiae (8 tibiae in each mouse group) were fixed in 70% ethanol, and the undecalcified bones were embedded in glycolmethacrylate. Sections 3 μm thick were cut longitudinally in the proximal region of the tibiae, and stained with toluidine blue. Histomorphometry was performed with a semiautomatic image analysis system (Histometry RT CAMERA, System Supply) linked to a light microscope. The histomorphometric measurements were made at ×400 using a minimum of 27-37 optical fields in the secondary spongiosa area at the growth plate-metaphyseal junction. The bone was labeled with calcein twice, with a 4-day interval between. The bone formation rate and mineral apposition rate were measured at the proximal tibia after calcein injection (16 mg/kg, Dojin). Nomenclature, symbols, and units are those recommended by the Nomenclature Committee of the American Society for Bone and Mineral Research [15].

### Blood chemistry

Blood was taken just before death, and the serum was prepared. The level of human and mouse adiponectin was measured using an ELISA kit for the human form (Otsuka) and mouse form (AdipoGen). Mouse insulin and leptin were determined using ELISA kits (Genzyme). Serum levels of RANKL, osteocalcin (OC), OPG and TRAP5b in serum were determined by ELISA with a mouse osteocalcin EIA kit (Biomedical Technologies), a mouse TRAP5b ELISA kit (Immunodiagnostic Systems), a mouse RANKL immunoassay kit (R&D Systems), and a mouse OPG immunoassay kit (R&D Systems), respectively. Assays were performed in accordance with the manufacturer's recommendation.

### Statistical analysis

All data are presented as mean ± S.D., and statistical analysis was performed by ANOVA with Dunnett adjustment. Differences were considered statistically significant at P < 0.05.

## Results

### Transgenic (Ad-Tg) mice overexpressing human full-length adiponectin in the liver

**Figure 1:**
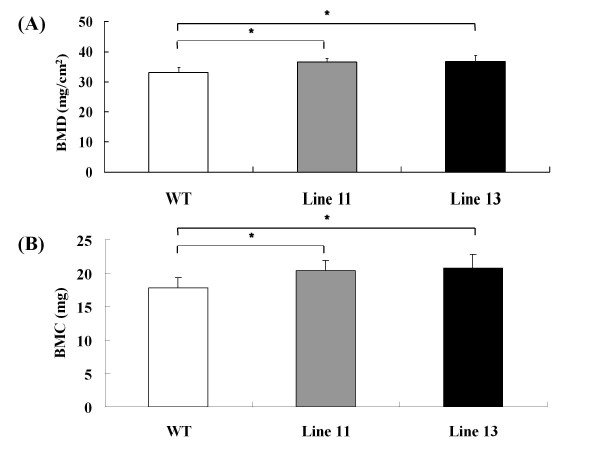
**Serum levels of human (A) and mouse (B) full-length adiponectin in 12-week-old Ad-Tg and WT littermate mice. **Data are expressed as means (bars) ± S.D. (error bar), n = 8. ND, not detected. Significant difference from WT serum level, P < 0.05.

To examine the endocrine effect of human adiponectin on bone metabolism, we analyzed two lines of 12-week-old male Ad-Tg mice overexpressing human adiponectin mRNA exclusively in the liver [14]. There was no human adiponectin expression in other tissue: muscle, visceral fat, spleen, or brain. The liver was histologically normal, with levels of hepatic enzymes similar to those in their WT littermates [14]. Ad-Tg mice in each line grew normally, were fertile, and appeared healthy with no gross histomorphological abnormalities. Measurement of the serum level of human and mouse full-length adiponectin in Ad-Tg mice using human and mouse adiponectin-specific ELISA demonstrated significant hyperadiponectinemia (Figure 1A, B). The plasma level was elevated in human adiponectin Tg mice, indicating a lack of negative feedback regulation. This finding had also been noted in our previous study [14]. Although the precise mechanisms responsible for the increase of endogenous mouse adiponectin in human adiponectin Tg mice was not clarified in the present study, it is conceivable that transgenic expression of adiponectin results in an increase in the total level of circulating adiponectin. The serum levels of leptin and insulin did not differ significantly between Ad-Tg mice and their WT littermates [14]. Thus, the Ad-Tg mice used in the present study were considered to be an appropriate model for characterizing the endocrine effect of human full-length adiponectin on bone metabolism.

### Serum levels of osteoblastic and osteoclastic markers

Firstly, we evaluated the levels of osteoblastic and osteoclastic markers in the serum of Ad-Tg and WT mice (Table 1). The serum osteocalcin (OC) level has long been used as a marker of osteoblast activity and new bone formation [16,17]. We therefore measured the level of OC in serum using a specific mouse OC ELISA to evaluate osteoblast activity in WT and Ad-Tg mice. The results showed that the serum OC level was significantly higher in the Ad-Tg mice than in their WT littermates, suggesting that osteoblastgenesis is increased in Ad-Tg mice. To evaluate osteoclast activity, the serum level of active TRAP5b, an enzyme that is released from osteoclasts during bone resorption [18], was examined and compared between Ad-Tg and WT mice. Unlike the serum OC level, the serum TRAP5b level demonstrated no significant difference between Ad-Tg and WT mice. To substantiate this further, we next measured the serum levels of RANKL and OPG, which are essential factors for osteoclast formation [19,20]. In accord with the results for TRAP5b, the serum levels of RANKL and OPG showed no significant difference between Ad-Tg and Wt mice.

**Table 1:** Serum levels of osteocalcin, TRAP5b, RANKL and OPG in 12-week-old Ad-Tg and WT littermate mice.

	WT	Ad-Tg Line11	Ad-Tg Line13
Osteocaltin (ng/ml)	25.2 ± 1.1	27.8 ± 0.8**	27.7 ± 0.9**
TRAP5b (U/L)	2.2 ± 0.4	2.1 ± 0.3	2.3 ± 0.4
RANKL (pg/ml)	68.6 ± 7.2	82.0 ± 24.6	77.1 ± 29.1
OPG (ng/ml)	2226.9 ± 61.4	2251.5 ± 212.0	2521.9 ± 277.8

Data are expressed as means ± S.D. (n = 8). **Significant difference from WT serum level, P < 0.05.

### Histomorphometric analysis of Ad-Tg mice

**Figure 2:**
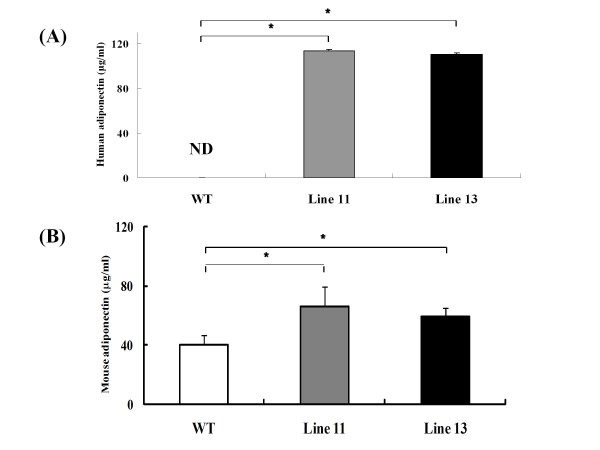
**BMD (A) and BMC (B) of the entire femur were measured by DEXA. **Data are expressed as means (bars) ± S.D. (error bar), n = 8. Significant difference from WT mice, P < 0.05.

To examine the role of endogenous human full-length adiponectin in bone metabolism, we analyzed the long bones of Ad-Tg mice and WT littermates at 12 weeks of age. BMD and BMC of the whole femur were consistently higher in Ad-Tg than in WT mice (Figure 2A, B).

Bone histomorphometric analysis was performed at the proximal tibiae in Ad-Tg and WT mice (Table 2). Bone volume was significanty increased due to the presence of human full-length adiponectin. Osteoblast surface and osteoid surface, both representative of the number of osteoblasts, were significantly greater in Ad-Tg mice than in their WT littermates. In contrast, bone resorption parameters including osteoclast number and eroded surface area, did not differ between the two types of mice. The mineral apposition rate, which reflects the bone formation ability of individual osteoblasts, was significantly higher in Ad-Tg mice (Table 2). Calcein double labeling showed that the width between the labeled sections in Ad-Tg mice was significantly increased than that in the WT littermates. Thus, Ad-Tg mice exhibited a high bone mass with increased bone formation but normal osteoclast function.

**Table 2:** Parameters for trabecular bone were measured below the growth plate at the proximal metaphysis of the tibia in Toluidine-Blue and calcein double-labeled sections.

	WT	Ad-Tg Line 11	Ad-Tg Line 13
BV/TV (%)	12.6 ± 2.7	16.1 ± 2.5*	17.3 ± 1.7**
Tb.N (/mm)	3.3 ± 0.3	3.9 ± 0.4*	3.9 ± 0.5*
OS/BS (%)	7.1 ± 4.1	17.7 ± 4.9**	15.4 ± 3.5*
O.Th (μm)	2.8 ± 0.2	3.3 ± 0.1**	2.8 ± 0.2
Ob.S/BS (%)	3.3 ± 2.8	7.4 ± 2.2*	9.3 ± 5.4**
N.Oc/B.Pm (/100 mm)	123.7 ± 41.1	112.2 ± 41.8	132.1 ± 60.7
Oc.S/BS (%)	1.0 ± 0.5	1.1 ± 0.7	1.4 ± 0.5
ES/BS (%)	2.7 ± 1.4	3.3 ± 1.5	4.4 ± 1.1
MAR (μm/day)	1.4 ± 0.2	1.7 ± 0.1**	1.6 ± 0.1*
MS/BS (%)	19.1 ± 3.2	33.1 ± 2.9**	26.8 ± 5.2*
BFR/BS (mm^3^/mm^2^/year)	0.09 ± 0.02	0.18 ± 0.03**	0.14 ± 0.03**
dLS/BS (%)	7.0 ± 1.0	20.7 ± 2.0**	14.6 ± 4.2**

BV/TV, bone volume/total tissue volume; Tb. N, trabecular number; OS/BS, osteoid surface/bone surface; O.Th, osteoid thickness; Ob.S/BS, osteoblast surface/bone surface; N.Oc/B.Pm, osteoclast number/bone perimeter; Oc.S/BS, osteoclast surface/bone surface; ES/BS, eroded surface/bone surface; MAR, mineral apposition rate; MS/BS (mineralization ratio), mineralizing surface/bone surface; BFR/BS, bone formation rate/bone surface; dLS/BS, double labeled surface/bone surface. Data are expressed as means ± S.D. of 8 bones/group. *P < 0.05, **P < 0.01, significantly different from WT mice.

## Discussion

A few studies using Ad-Tg mice have assessed the role of adiponectin in bone metabolism, but the results have been conflicting [8,10,21]. Oshima et al. demonstrated that transient overexpression of full-length mouse adiponectin for 2 week by adenoviral infection in 8-week-old C57BL/6J mice increased trabecular bone volume in the distal femur compared with LacZ overexpressing controls, with reduced number of osteoclasts in TRAP-stained sections [8]. These results concurred with our data in view of increased bone mass; however, they did not report the serum levels of adiponectin, insulin, and body weights. Shinoda *et al. *demonstrated no significant differences in bone mass or turnover in 8-week-old male Ad-Tg mice overexpressing globular-type adiponectin specifically in the liver, compared to their WT littermates [10]. In their study, no differences in BMD of the femur, tibia, and vertebrae and no differences in bone formation or resorption parameters were found in Ad-Tg mice [10]. Recent studies have demonstrated that the biological effect of adiponectin depends on its molecular structure [22,23], suggesting that the full effects of adiponectin on bone may be mediated by full-length adiponectin, rather than the globular type. Another study has demonstrated that female Ad-Tg mice had a significantly lower bone mineral content at 8 and 16 weeks of age, and that the femur neck peak load was significantly lower in 8-week-old Ad-Tg mice of both genders, in comparison with controls [21]. They concluded that circulating adiponectin is a negative regulator of bone mineral and bone strength in mice. For their study, they used transgenic mice expressing a dominant mutation in the collagenous domain of adiponectin, resulting in an increased level of adiponectin complexes including full-length adiponectin; there was also a sexual dimorphism in that females had significantly higher circulating levels than males [21].

Two groups have previously reported the skeletal changes in Ad KO mice. Shinoda et al. found no effect on bone phenotype in Ad-knockout mice at 8 weeks of age [10]. Williams et al. reported increases in the number of trabeculae and bone volume of the proximal tibia at 14 week of age, but non-significant trends at 8 and 22 weeks, in Ad-knockout mice [24]. Interpreted broadly, these studies gave similar results: wild-type and Ad-knockout mice are similar in early life, but differences emerge in adulthood. Most investigators have consistently demonstrated that Ad-knockout mice have either spontaneous or diet-induced insulin resistance, with hyperinsulinemia [25,26]. Thus, insulin resistance is one potential explanation for the increased bone mass in Ad-knockout mice.

The present study utilized 12-week-old male mice overexpressing human full-length adiponectin specifically in the liver and focused on the endocrine effects of this molecule on bone, since human adiponectin is biologically active in mice [14,27]. A noteworthy finding was that circulating human full-length adiponectin increased bone mass and BMD in Ad-Tg mice by promoting osteoblast formation while osteoclast number remained normal. To our knowledge, similar findings have not been reported previously.

According to the recent in vitro studies [8,10], we have demonstrated that circulating human full-length adiponectin increased bone formation in mice. In our previous study, hyperadiponectinemia in the transgenic mice resulted in suppression of fat accumulation and prevention of premature death by a high-calorie diet, leading to an increase of insulin sensitivity [14]. It is therefore considered that the increased osteoblastgenesis may have resulted from the anabolic effects of insulin on bone. In addition, an increase in osteoblast number could well be the result of decreased oxidative stress that attenuates the apoptosis of pre-existing osteoblasts [28]. The latter interpretation is consistent with the evidence discussed previously regarding the phenotype of age-related/Ad KO mice with altered adiponectin expression.

In the present study, circulating human full-length adiponectin did not affect the number of osteoclasts. There was no significant difference in serum RANKL/OPG levels between Ad-Tg mice and their WT littermates. Bone histomorphometric and immunohistochemical analysis consistently demonstrated normal osteoclast function in Ad-Tg mice. As mentioned above, increased insulin sensitivity was thought to be an important factor for skeletal phenotype in our transgenic mice. The major substrates of insulin are known to be closely related insulin receptor substrate-1 and -2: IRS-1 and -2 [29]. Osteoclastic cells showed IRS-2 expression, but not IRS-1 expression [30]. Intrinsic IRS-2 in osteoclastic cells was not important for their differentiation, function, or survival despite the fact that IRS-2 and receptors of insulin are expressed in these cells [31,32]. Thus, these observations may explain why the circulating human full-length adiponectin did not influence the number of osteoclast in the transgenic mice.

## Conclusions

We have demonstrated that elevation of the circulating adiponectin level increases bone mass by activating bone formation while normal osteoclast function is retained. Our study confirms that hyperadiponectinemia positively regulates osteogenesis, thus enhancing bone formation.

## Abbreviations

Ad-Tg mice: Adiponectin Transgenic mice; WT: Wild Type; BMD: Bone Mineral Density; BMC: Bone Mineral Content; OC: Osteocalcin; IRS: Insulin Receptor Substrate

## Competing interests

The authors declare that they have no competing interests.

## Authors' contributions

YM, MG and NF designed the study and drafted the manuscript. YM, NF, IS, SO, XY, TH, NW, AM, TY and SY performed the experimental work and the statistical analysis. KY and KN participated in study design. All authors have read and approved the final manuscript.

## Pre-publication history

The pre-publication history for this paper can be accessed here:

http://www.biomedcentral.com/1471-2474/12/18/prepub
